# Identification of a new circulating recombinant form of human immunodeficiency virus type 1, CRF124_cpx involving subtypes A, G, H, and CRF27_cpx in Angola

**DOI:** 10.3389/fmicb.2022.992640

**Published:** 2022-10-17

**Authors:** Rayana Katylin Mendes Da Silva, Joana Morais, Brian Thomas Foley, Gonzalo Bello, Mariza Gonçalves Morgado, Monick Lindenmeyer Guimarães

**Affiliations:** ^1^Laboratório de AIDS e Imunologia Molecular, Instituto Oswaldo Cruz, FIOCRUZ, Rio de Janeiro, Brazil; ^2^Laboratório de Biologia Molecular, Instituto Nacional de Investigação em Saúde, Ministério da Saúde de Angola, Luanda, Angola; ^3^Departamento de Bioquímica, Faculdade de Medicina, Universidade Agostinho Neto, Luanda, Angola; ^4^Theoretical Biology and Biophysics, Los Alamos National Laboratory, Los Alamos, NM, United States

**Keywords:** HIV-1, diversity, CRF124_cpx, surveillance, Angola

## Abstract

Angola, located in Central Africa, has around 320,000 (270,000–380,000) people living with human immunodeficiency virus (HIV)/AIDS, equivalent to 1% of the country’s population at the end of 2021. A previous study conducted in 2012, using Angolan samples collected between 2008 and 2010 revealed a high prevalence of HIV-1 recombinants, around 42% of sequences, with 21% showing the same UH profile in partial pol region which were grouped into a monophyletic cluster with high bootstrap support. Thus, the objective of the present work was to obtain complete genomes of those sequences and characterize them, aiming at a description of a new circulating recombinant form (CRF). Whole blood from nine HIV-1 UH *pol*-infected individuals had their genomic DNA extracted, and nested PCR was used to amplify seven overlapping fragments targeting the full-length HIV-1 genome. The final classification was based on maximum likelihood trees, and recombination analyses were performed using a bootscan from the Simplot program. BLAST and Los Alamos Database inspections were used to search other similar H-like *pol* sequences. Complete genome amplification was possible for three samples, partial genomes were obtained for the other three, and only *pol* was available for the remaining three sequences. Bootscan analysis of the two whole-genome and three partial genome sequences retrieved from people living with HIV/AIDS (PLHIVA) without epidemiological linkage showed the same complex recombination profile involving HIV-1 subtypes A/G/H/CRF27_cpx, with a total of six recombinant breakpoints, aiming to classify a new HIV-1 CRF124_cpx. We found no other full-length HIV-1 genomes with the same mosaic profile; however, we identified 33 partial *pol* sequences, mainly sampled from Angola between 2001 to 2019, with the same H-like profile. Bayesian analysis of H and H-like *pol* sequences indicates that CRF124_cpx probably originated in Angola at mid-1970s, indicating that this CRF has been circulating in the country for a long time. In summary, our study describes a new CRF circulating principally in Angola and highlights the importance of continuing molecular surveillance studies, especially in countries with high molecular diversity of HIV.

## Introduction

About 40 years after its discovery, the human immunodeficiency virus (HIV) remains a global public health challenge. By the end of 2021, 38.4 million people were living with HIV/AIDS (PLWHA) worldwide (UNAIDS, 2022).

Human immunodeficiency virus is classified into two types (type 1 and type 2). HIV-1 is made up of four groups (M, N, O, and P), while HIV-2 is made up of eight groups (A–H; Salvi et al., 1998; Simon et al., 1998; Heyndrickx et al., 2000; Robertson, 2000; Guimarães et al., 2002; Roques et al., 2004; Plantier et al., 2009). HIV-1 group M is responsible for the HIV/AIDS pandemic. Full-genome phylogenetic analyzes have revealed that this group is subdivided into 10 subtypes (A–D, F–H, and J–L) and nine sub-subtypes (A1–A4, A6–A8, F1, and F2). In addition to more than 100 CRFs and uncounted unique recombinant forms (URFs; Désiré et al., 2018; Yamaguchi et al., 2020; Mendes Da Silva et al., 2021; Los Alamos database, 2022).

Overall, on the African continent, the most remarkable diversity of HIV is found in the Central African region, where HIV-1 originated, and all subtypes and many CRFs and URFs are verified (Hemelaar et al., 2019). Like other countries in this region, Angola has high genetic variability of HIV-1 in its population, with an estimated 320,000 (270,000–380,000) PLHIVA, equivalent to 1% of the country’s population until the end of 2021 (UNAIDS, 2021). In a previous study of HIV samples from Angola, molecular epidemiological data of the pol region revealed a high prevalence of HIV-1 recombinant forms (42%), subtypes C (16%) and F1 (14%), followed by G (6%), A, D, and H (5% each), and K (1%) (Delwart et al., 1993; Delatorre et al., 2017). Among the 42% of the sequences classified as recombinants, 21% had the same UH *pol* profile and were grouped in a monophyletic cluster with high support (Passaes et al., 2009; Afonso et al., 2012a). Thus, the present work aimed to obtain and characterize the HIV-1 full-length genome sequences, aiming to describe a new CRF.

## Materials and methods

### Study population

Previous studies from our group recruited 101 PLHIVA in Angola, and none of them were under the regular antiretroviral therapy. The study was approved by the CE-FMUAN 027/08 Ethical Committee. After signing the informed consent, patient’s blood collections were carried out in three phases from July 2008 to November 2010 (August 2008, July 2009, and November 2010) at São Lucas Medical Center (CSSL), one of the National Referral Centers for HIV diagnoses, in Kifangondo village, located in the border between Luanda and Bengo provinces. HIV-1 *pol* sequences [covering the protease (PR) and partial reverse-transcriptase (RT), positions 2,313–3,272 relative to the HXB2 genome] were generated. A highly significant supported UH *pol* cluster of nine sequences, that corresponded to 8.9% of the total HIV sequences analyzed (Qu et al., 2010; Afonso et al., 2012a), were the focus of the present study.

### Amplification of HIV-1 full-length genomes and phylogenetic analyses

Biological samples were stored at −20°C since collection in 2008/2010. DNA was extracted using the QIAamp DNA Blood Mini Kit (QIAGEN, Germany) or QIAmp Viral RNA (QIAGEN, Germany). The double-stranded proviral DNA was amplified using nested-PCR employing Platinum Taq DNA polymerase (Invitrogen, Carlsbad, CA, United States) into seven overlapping fragments using HIV-1 specific primers, as presented in Supplementary Table S1 (Zazzi et al., 1993).

The amplified products were purified using the Illustra GFX PCR DNA and gel Purification Kit (GE Healthcare, Little Chalfont, Buckinghamshire, United Kingdom) and sequenced on an ABI 3130 Genetic Analyzer using the ABI BigDye Terminator v.3.1 Cycle Sequencing Ready Reaction kit (Applied Biosystems, Foster City, CA, United States). The chromatograms were analyzed and edited using the Seqman software package DNASTAR Lasergene (DNAStar, Madison, WI, United States).

The phylogenetic trees of maximum likelihood (ML) were reconstructed with PhyML version 3.0 (Guindon et al., 2010) using the general time-reversible (GTR) model of nucleotide substitutions. The approximate likelihood ratio test (aLRT) was used to estimate the confidence of the branch in the tree. The phylogenetic trees reconstructed were visualized and edited using the Figtree software version 1.4.4 [13]. Reference sequences of HIV-1 group M subtypes (A–D, F–H, and J–L), sub-subtypes (A1–A4, A6–A8, F1, and F2), and all CRF (until CRF118) sequences were obtained from the Los Alamos HIV database (Available from: http://www.hiv.lanl.gov/).

Recombination analyses were performed using a Bootscan implemented in SimPlot v3.5.1 software with the following parameters: 400 nucleotides (nt) window, 20 nt increments, and NJ method under Kimura’s two-parameter correction with 100 bootstrap replicates (Lole et al., 1999). For better characterization of recombination breakpoints, the midpoints were mapped as suggested in previous analyzes performed by Sierra et al. (2005).

A basic local alignment search tool (BLAST, Available at: https://blast.ncbi.nlm.nih.gov) was performed to identify sequences with high similarity to the studied sequences. The retrieved sequences, together with those from HIV-1 subtype H or UH from Los Alamos Database, were used to search other H-like *pol* sequences and were included in phylogenetic analyses. (available from: https://blast.ncbi.nlm.nih.gov; http://www.hiv.lanl.gov).

### Sequence availability

All HIV-1 sequences generated in this study were deposited in the GenBank database (accession numbers ON962802–ON962807).

### Analysis of spatiotemporal dispersion pattern and demographic history

The evolutionary rate (*μ*, nucleotide substitutions per site per year, subst./site/year), the time of the most recent common ancestor (T_MRCA_, years), and the ancestral geographic movements of HIV-1 H and H-like *pol* sequences were jointly estimated using the Bayesian Markov Chain Monte Carlo (MCMC) approach as implemented in BEAST v1.10 (Drummond et al., 2002; Drummond and Rambaut, 2007) with BEAGLE to improve run-time (Suchard and Rambaut, 2009). Analyses were performed under a GTR + I + G nucleotide substitution model, a relaxed uncorrelated lognormal molecular clock model with a uniform prior on clock rate (1.0–2.0 × 10^−3^ subst/site/year), and a Bayesian Skyline coalescent model (Drummond et al., 2005). Migration events throughout the phylogenetic history were inferred using a reversible discrete Bayesian phylogeographic model (Lemey et al., 2009) and a CTMC rate reference prior (Ferreira and Suchard, 2008). MCMC chain was run for 10 × 10^6^ generations and adequate chain mixing (Effective Sample Size > 200) and uncertainty in parameter estimates [95% Highest Probability Density (HPD) interval] were assessed using the TRACER v1.6 program (Rambaut and Drummond, 2009). Maximum clade credibility (MCC) tree was summarized from the posterior distribution of trees with Tree Annotator and visualized with FigTree v1.4.0 (Available from: http://tree.bio.ed.ac.uk/software/figtree/).

## Results

### Sociodemographic and clinical data

Among the nine sequences previously classified as HIV-1 UH investigated in PLWHA from Angola, seven were from provinces bordering the CSSL, and two were from the province of Cabinda and Uíge (Northern Angola). Seven were female, and two were male. Sociodemographic and clinical data of our study group are presented in Table 1. The mean age of these individuals was 34 (24–36) years at the time of sample collection. Only two patients had a known epidemiological linkage to sexual transmission (ANG. 107e ANG.113). None of the patients were under antiretroviral therapy (ART). However, some patients reported being part of a spiritual group where the chief prescribed some natural herbs and medicines for treatment (Afonso et al., 2012a).

**Table 1:** Sociodemographic and clinical data of the studied individuals.

Sample	Gender	Age	Province	Year of Diagnosis	Treatment Status	Year of ARV start
ANG.05	F	24	Luanda	2006	-	-
ANG.37	F	42	Cuanza Sul	2007	Naive	-
ANG.44	F	36	Bengo	2007	Treated^*^	2009
ANG.53	F	34	Cabinda	2008	Naive	-
ANG.71	M	34	Uíge	2005	Naive	-
ANG.75	F	28	Luanda	2005	Naive	-
ANG.78	F	42	Cuanza Norte	2005	Naive	-
ANG.107	M	34	Bengo	2004	Treated^*^	2008
ANG.113	F	32	Bengo	2004	Treated^*^	2008

* Alternative treatment; F, Female; and M, Male.

### Genome amplification and sequence analysis

From these previously classified HIV-1 UH *pol* samples, it was possible to amplify and sequence the complete genome of three of them (ANG.37, ANG.44, and ANG.78), obtained from PLWHA without epidemiological linkage. Due to the low amount of the biological material available, only partial genome sequences could be obtained for three individuals (ANG.05, ANG.71, and ANG.113), and, for the three remaining ones, only the initial fragment of PR/RT was obtained from the original study and investigated (Table 2).

**Table 2:** Genomic data related to the studied HIV Angolan sequences.

Sample	Year of Collection	Position relative to the HXB2 genome	Classification	GenBank Accession
ANG.05	2008	407–5,915	CRF124_cpx^*^	ON962805
ANG.37	2009	406–9,613	URF^**^	ON962802
ANG.44	2009	471–9,509	CRF124_cpx^*^	ON962803
ANG.53	2009	2,313–3,272	UH	JN937066
ANG.71	2009	2,214–5,662	CRF124_cpx^*^	ON962806
ANG.75	2009	2,313–3,272	UH	JN937087
ANG.78	2009	878–9,512	CRF124_cpx^*^	ON962804
ANG.107	2009	2,313–3,272	UH	JN937112
ANG.113	2009	857–5,662	CRF124_cpx^*^	ON962807

* A (878–1,369), G (1,370–2,241), H (2,242–4,212), CRF27_cpx (4,213–6,074), H (6,075–8,160), G (8,161–8,887), and H (8,888–9,496 relative to the HXB2 genome).

** A7 (878–1,369), CRF124_cpx (1,370–8,887), and J (8,888–9,496 relative to the HXB2 genome).

**Figure 1:**
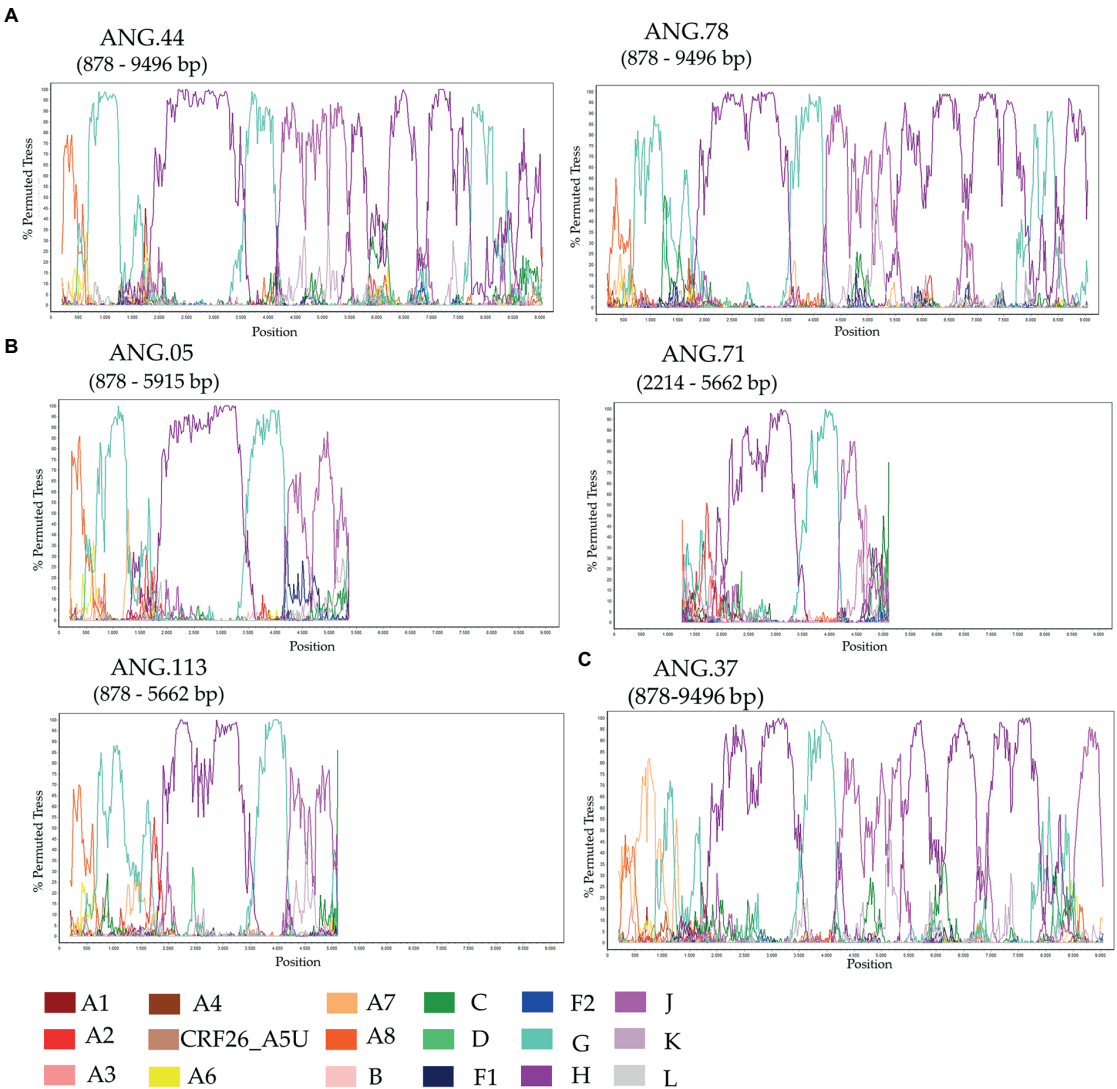
Investigation of human immunodeficiency virus (HIV) genome recombination in HIV-1 samples from Angola. Bootscan analysis was performed in SimPlot software using a 400 nt sliding window and 20 nt increments. **(A)** Complete genome (ANG.44 and ANG.78), **(B)** Partial genome (ANG.05, ANG.71, and ANG.113), and **(C)** ANG.37. The legend in the lower corner indicates the colors that represent the different clades of HIV-1.

The ML tree performed confirmed that the sequences ANG.37, ANG.44, and ANG.78 grouped apart of the remaining HIV-1 subtypes and CRFs, suggesting a new subtype or CRF (Supplementary Figure 1). Bootscan analysis, including all HIV-1 group M subtypes and A and F sub-subtypes was conducted for the full-length genome sequences (Figure 1A) and partial genome sequences (Figure 1B). This analysis revealed that five of the six sequences (Figures 1A,B) showed the same recombination profile, involving HIV-1 subtypes A, G, H, and J, with eight recombinant breakpoints. In order to confirm these breakpoints and the HIV-1 subtype of each fragment, partial ML phylogenetic trees were performed (Supplementary Figure 2). The sequence ANG.37 showed the same recombinant pattern as ANG.44 and ANG.78, from the fragment positions 1,370 to 8,887, depicting a distinct recombination profile outside this fragment (Figure 1C), showing a URF profile.

**Figure 2:**
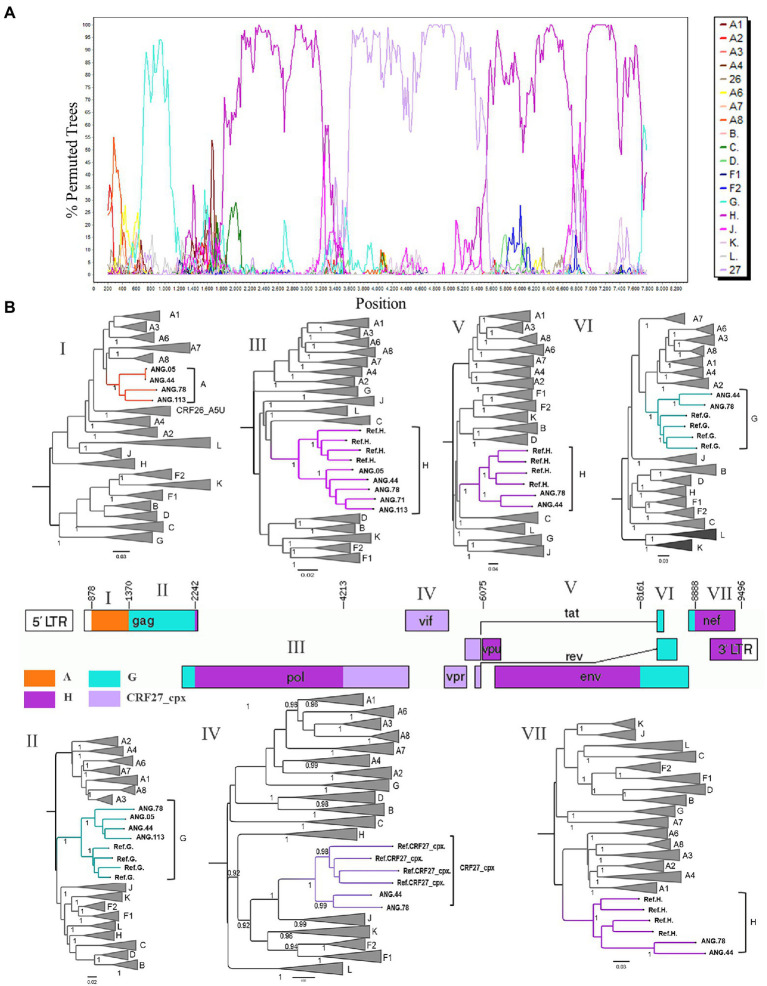
Determination of the new circulating recombinant form (CRF) among HIV-1 samples circulating in Angola. **(A)** Bootscan analysis of the sequence ANG.44 was performed in SimPlot software using a 400 nt sliding window and 20 nt increments. **(B)** Genomic structure of CRF124_cpx colored according to the HIV-1 subtyping. The mosaic map was generated using the Recombinant HIV-1 Drawing Tool (https://www.hiv.lanl.gov/content/sequence/DRAW_CRF/recom_mapper.html). The maximum likelihood tree (ML) was performed to confirmation of the HIV-1 subtype of each fragment. ML tree implementing nucleotide substitution model General Time Reversible (GTR), indicating the phylogenetic relationships between pure HIV-1 subtypes and CRF124_cpx sequences. ALRT values were represented only if >0.90.

After observing that ANG.44 and ANG.78 sequences cluster together with the CRF13_cpx, CRF18_cpx, and CRF27_cpx, all of them involved the HIV-1 subtypes A, G, J, and H (except CRF13_cpx), we have included their full-length genome sequences to perform a new bootscan analysis. Through bootscanning it was possible to verify that the fragments IV (subtype G) and V (subtype J), represented at Supplementary Figure 2, should be reclassified in CRF27_cpx (4213–6,075 relative position to HXB2 genome; Figure 2). Based on these analyzes and in the lack of epidemiological linked among this PLHIVA, we could describe a new CRF, designated as CRF124_cpx, depicting six recombinant breakpoints and involving HIV-1 subtypes A, G, H, and CRF27_cpx (Table 2).

**Figure 3:**
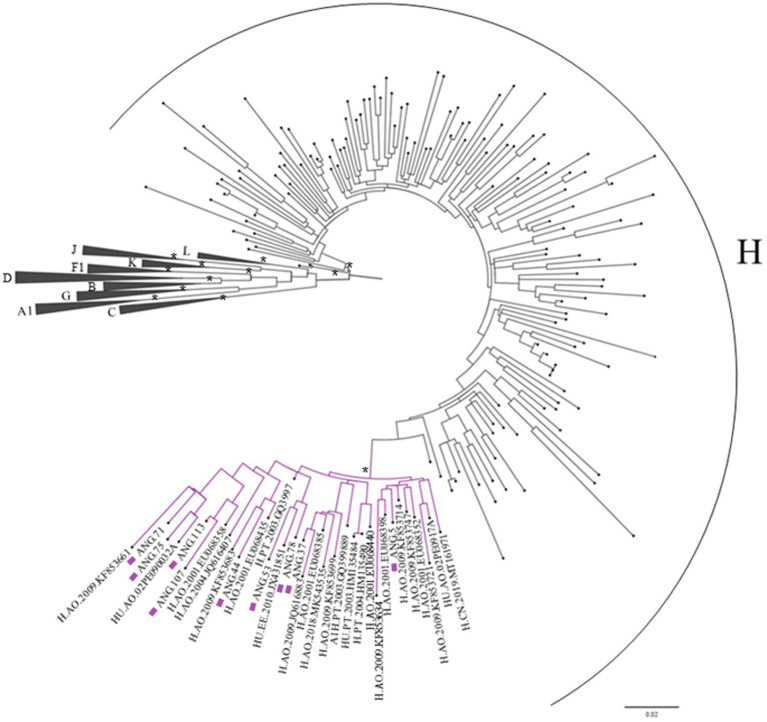
Maximum likelihood (ML) tree of Pol fragment (2,261–3,274 relative to the HXB2 reference) implementing nucleotide substitution model General Time Reversible (GTR), indicating the phylogenetic relationships between the studied sequences and those retrieved from BLAST. Approximate likelihood ratio test (ALRT) values are represented only if >0.90 with asterisk. The sequences belonging to the CRF124_cpx are depicted with purple squares and their cluster are highlighted in purple.

Complete genome sequences and all seven fragments that compose the CRF124_cpx were submitted to BLAST to scan for related sequences and did not recover significantly related sequences. Thus, we performed BLAST analyses of partial *pol* (2,261–3,274) and retrieved 100 sequences with homology higher than 91.4%. These retrieved sequences and those subtyped as H, previously published and available in the database at Los Alamos HIV database were included in this alignment, and the ML tree was performed. This analysis allowed us to identify another 24 sequences that presented the same H-like profile defined by bootscan analysis, showing part of the segment with threshold below 70 and another part well defined as H, and all these sequences clustered together with high support (aLTR 0.99) with CRF124_cpx in this fragment, as highlighted in purple in Figure 3.

### Analysis of spatiotemporal dispersion pattern and demographic history

**Figure 4:**
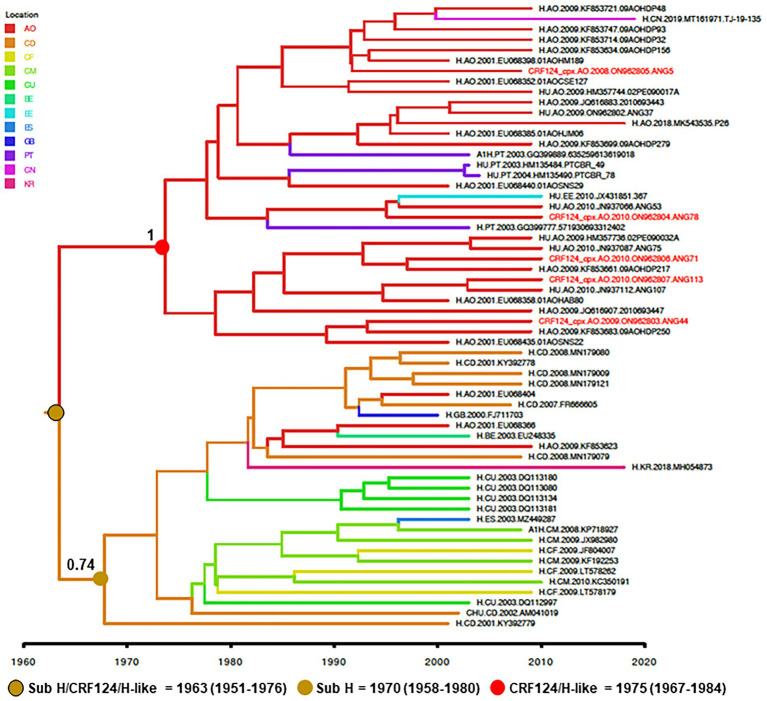
Time-scaled Bayesian MCC tree of HIV-1 CRF124_cpx, H and H-like *pol* sequences (~700 nt). Branches are colored according to the most probable location state of their descendent nodes as indicated in the legend (upper left). Red tips indicate the position of full-length CRF124_cpx sequences. *PP* support is shown at nodes of subtype H and clade CRF124_cpx/H-like. Branch lengths are drawn to a scale of years. The tree was automatically rooted under the assumption of a relaxed molecular clock. AO, Angola; CD, Democratic Republic of Congo; *CF*, Central African Republic; CM, Cameroon; CU, Cuba; BE, Belgium; EE, Estonia; ES, Spain; GB, Great Britain; PT, Portugal CN, China; and KR, South Korea.

To reconstruct the origin of the HIV-1 CRF124_cpx, the two full-length and three partial CRF124_cpx sequences here characterized were combined with 28 H-like and 27 subtype H *pol* sequences sampled between 2000 and 2019, retrieved from Los Alamos HIV database, and subjected to Bayesian phylogeographic analysis. Our analysis indicates that the CRF124_cpx/H-like and subtype H *pol* sequences branched in two highly supported (posterior probability, *PP* = 1 and *PP* = 0.74, respectively) reciprocally monophyletic sister clades, with full-length CRF124_cpx sequences widely intermixed among the H-like *pol* sequences (Figure 4). The ancestor of the H and CRF124_cpx/H-like clades was placed in the Democratic Republic of Congo (posterior state probability, *PSP* = 0.60) at 1963 (95% HPD, 1951–1976; Figure 4). The most probable root location of the CRF124_cpx/H-like clade was placed in Angola (posterior state probability, *PSP* = 1) and the T_MRCA_ was estimated at 1975 (95% HPD, 1967–1984), while the subtype H ancestor was placed in the Democratic Republic of Congo (posterior state probability, *PSP* = 0.79) at 1970 (95% HPD, 1958–1980; Figure 4). The subtype H was disseminated from the Democratic Republic of Congo to other Central African countries (Angola, Cameroon, and Central African Republic) and to other countries around the world, while the CRF124_cpx/H-like clade migrates from Angola to Portugal, Estonia and China at multiple independent times.

## Discussion

Angola is a Central Africa country that borders the Republic of Congo, Democratic Republic of Congo, Zambia, and Namibia. As previously described the HIV-1 epidemic in Angola is highly complex; all pure non-B pure subtypes belonging to group M have already been identified, in addition to the high proportion of URFs and CRFs (Bártolo et al., 2009; Afonso et al., 2012a; Sebastião et al., 2020). As compiled by Hemelaar et al. in 2019, the prevalence of recombinant forms increased from 16.5% (1990–1999) to 46.8% (2010–2015) in this African region (Hemelaar et al., 2019). Through HIV molecular epidemiology studies carried out between 2000–2009 and 2010–2019, it was also possible to observe an increasing number of recombinant sequences from 23.6 to 41.4% in Angola in these periods. Among the sequences with a H-like profile in the *pol- PR/RT* region, it was possible to stand out that sequences presenting the same profile have already been reported in five of eight published studies, totalizing 20 sequences. Based on the 448 HIV-1 Angolan sequences (positions 2,661 –3,274 relative to HXB2), this profile, H-like detected in 28 Angolan sequences corresponds to 6.2% of the deposited sequences (Bártolo et al., 2009, 2014; Castelbranco et al., 2010; Clemente, 2011; Afonso et al., 2012a; Sebastião et al., 2020; Los Alamos database, 2022).

Recombinants are composed of segments of different combinations between subtypes or CRFs, potentially giving viruses an evolutionary advantage as enhanced transmission, pathogenesis, or drug resistance. Some recombinants are so successful in spreading that they become more prevalent than some pure HIV-1 subtypes, such as the worldwide prevalence of CRF02_AG compared to subtype K, 7.7 and 0.1%, respectively (Hemelaar et al., 2020; Los Alamos database, 2022). In the present study, it was possible to characterize a new circulating recombinant form, called CRF124_cpx, composed of fragments of subtypes A, G, H, and CRF27_cpx. After the change in the nomenclature proposed to the HIV classification based on genomes which included the criterion adopted for the description of new CRFs, proposed for Robertson in 2000, over the years, more than 100 CRFs have been identified. CRFs involving subtypes A, G, H, and J do not appear to be uncommon; about 12 CRFs already been described were composed by the association of at least two of the subtypes involved in this newly described (Los Alamos database, 2022). By inspecting complete genomic sequences in the Los Alamos Database, it was possible to identify that HIV-1 subtype A is mainly found in Rwanda and Russian Federation; subtype G in Nigeria and Cameroon; subtype H in the Democratic Republic of Congo and Belgium; and subtype J is more detected in sequences from Sweden and Democratic Republic of Congo, being the Democratic Republic of Congo one of the countries that border Angola (Supplementary Table S2). Interestingly, the HIV-1 sub-subtype A detected in fragment I (878–1,370 from HXB2 genome) from the CRF124_cpx is distinct from the other A sub-subtype, suggesting that it took part in a new one not already described.

Different studies have been carried out to analyze the molecular epidemiology of HIV-1 in Angola, based in the partial pol region, and the HIV-1 subtypes A, G, H, and J, which comprise CRF124_cpx, were identified in their pure forms. In these studies, the HIV-1 subtype A prevalence ranged from 5 to 13.8%, subtype G from 5.7 to 10.8%, subtype H from 3.8 to 5.7% and J was not found in one study and was found 3.2% in another (Bártolo et al., 2009, 2014; Afonso et al., 2012a; Sebastião et al., 2019). The co-circulation of these subtypes in Angola may have favored the occurrence of recombination events that gave rise to the CRF124_cpx. It is also possible that the CRF124_cpx ancestor was disseminated from the neighboring country of Democratic Republic of Congo to Angola. Our Bayesian phylogeographic analysis supports that the CRF124_cpx/H-like *pol* sequences branched as a sister clade of subtype H and that the ancestor of both lineages probably arose in the Democratic Republic of Congo in the early 1960s. The subtype H probably started to spread in the Democratic Republic of Congo at the early 1970s and was disseminated to neighboring Central African countries (including Angola, Cameroon, and the Central African Republic). The CRF124_cpx/H-like lineage started to spread in Angola around the mid-1970s and was not detected in other Central African countries. Of note, the CRF27_cpx that comprise fragment 4,213–6,075 (relative to the HXB2 genome) of the CRF124_cpx genomes is detected at low prevalence (0.75%) in the Democratic Republic of Congo (Vidal et al., 2008). We may thus speculate that the H-like ancestor may represents a H sub-subtype that circulated in the Democratic Republic of Congo between 1960 and 1980 and that became extinct as a pure sub-subtype, but recombined with other HIV-1 group M subtypes to originate the CRF124_cpx and others URFs.

The inferred T_MRCA_ of the CRF124_cpx clade overlap with the estimated T_MRCA_ of the subtype F1 clade (Bello et al., 2012) and of some local subtype C clades (Afonso et al., 2012b) in Angola. Of note, the concurrent onset date of HIV-1 clades C, F1, and CRF124_cpx in Angola between the mid-1970s at the early 1980s coincides with important socio-political changes that occurred in the country after the beginning of the civil war in 1975 and also coincides with a period of positive migration influx for Angola according to the estimates of the United Nations World Population Prospects (available from: http://esa.un.org/unpd/wpp/Excel-Data/migration.htm). Those changing migratory patterns between mid-1970s and early 1980s may have fueled the introduction, recombination, and local dissemination of different HIV-1 viruses in Angola. The search for sequences presenting the same *pol* profile of this new CRF124_cpx demonstrates that it is not restricted to Angola, but have been probably disseminated to Portugal, China, and Estonia. Some studies have shown a substantial similarity in the HIV-1 molecular epidemiology profile between Angola and Portugal. Their hypothesis is that socio-historical ties and the intense human migration between the 1970s and 1980s, added to the current intense migratory flow between Portugal and Sub-Saharan African countries, mainly those of the Portuguese-speaking African countries, such as Angola, contributed to the introduction of non-B HIV-1 subtypes in Portugal (Bártolo et al., 2009; Niyasom et al., 2009; Sebastião et al., 2020).

The diversity of HIV can impact diagnosis, viral load measurement, drug resistance, responses to antiretroviral treatment, pathogenesis, vaccine design, immune response, and viral escape (Mackay et al., 2004; Hemelaar, 2013), which demonstrates the need for continuous monitoring of the molecular epidemiology of HIV worldwide.

## Data availability statement

The data presented in the study are deposited in the GenBank database repository, accession number ON962802–ON962807.

## Ethics statement

The studies involving human participants were reviewed and approved by CE-FMUAN 027/08 Ethical Committee. The patients/participants provided their written informed consent to participate in this study.

## Author contributions

MG, MM, and JM conceived and designed the study. RS, JM, GB, and MG performed the experiments. JM participated in patient recruitment. RS, MG, GB, and BF analyzed the data. MG and RS drafted the manuscript. All authors contributed to the article and approved the submitted version.

## Funding

We received financial support from Conselho Nacional de Desenvolvimento Científico e Tecnológico/CNPq (Grant number: 307972/2014-3) and from the Laboratory of AIDS and Molecular Immunology, Oswaldo Cruz Institute, FIOCRUZ. RS, MM, and MG are recipients of fellowships from CNPq, numbers 132017/2020-2, 314064/2018-4, and 305919/2018-0, respectively. GB is funded by fellowships of CNPq (Grant number: 304883/2020-4) and FAPERJ (Grant number: E-26/202.896/2018). This study was partially supported by the Coordenação de Aperfeiçoamento de Pessoal de Nível Superior - CAPES - Finance Code 001.

## Conflict of interest

The authors declare that the research was conducted in the absence of any commercial or financial relationships that could be construed as a potential conflict of interest.

## Publisher’s note

All claims expressed in this article are solely those of the authors and do not necessarily represent those of their affiliated organizations, or those of the publisher, the editors and the reviewers. Any product that may be evaluated in this article, or claim that may be made by its manufacturer, is not guaranteed or endorsed by the publisher.
