# Supplementary figures and images for: Identification of a new circulating recombinant form of human immunodeficiency virus type 1, CRF124_cpx involving subtypes A, G, H, and CRF27_cpx in Angola
Source: Front Microbiol. 2022 Oct 17;13:992640. doi: 10.3389/fmicb.2022.992640 (PMC9619209; doi:10.3389/fmicb.2022.992640)

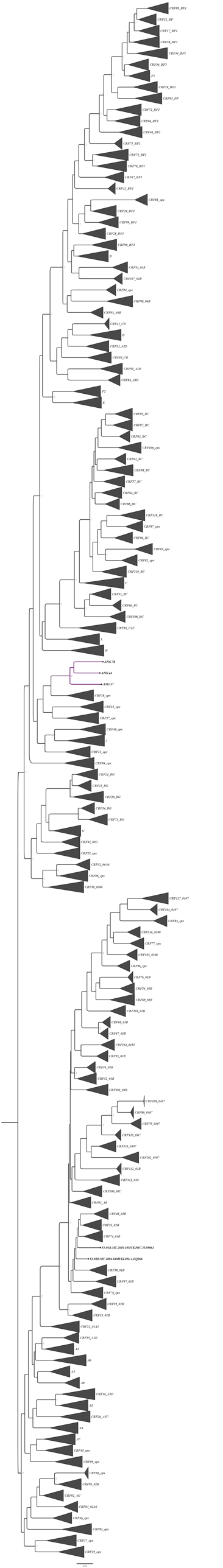

Supplement: Supplementary file 1 [file Image_1.TIF]

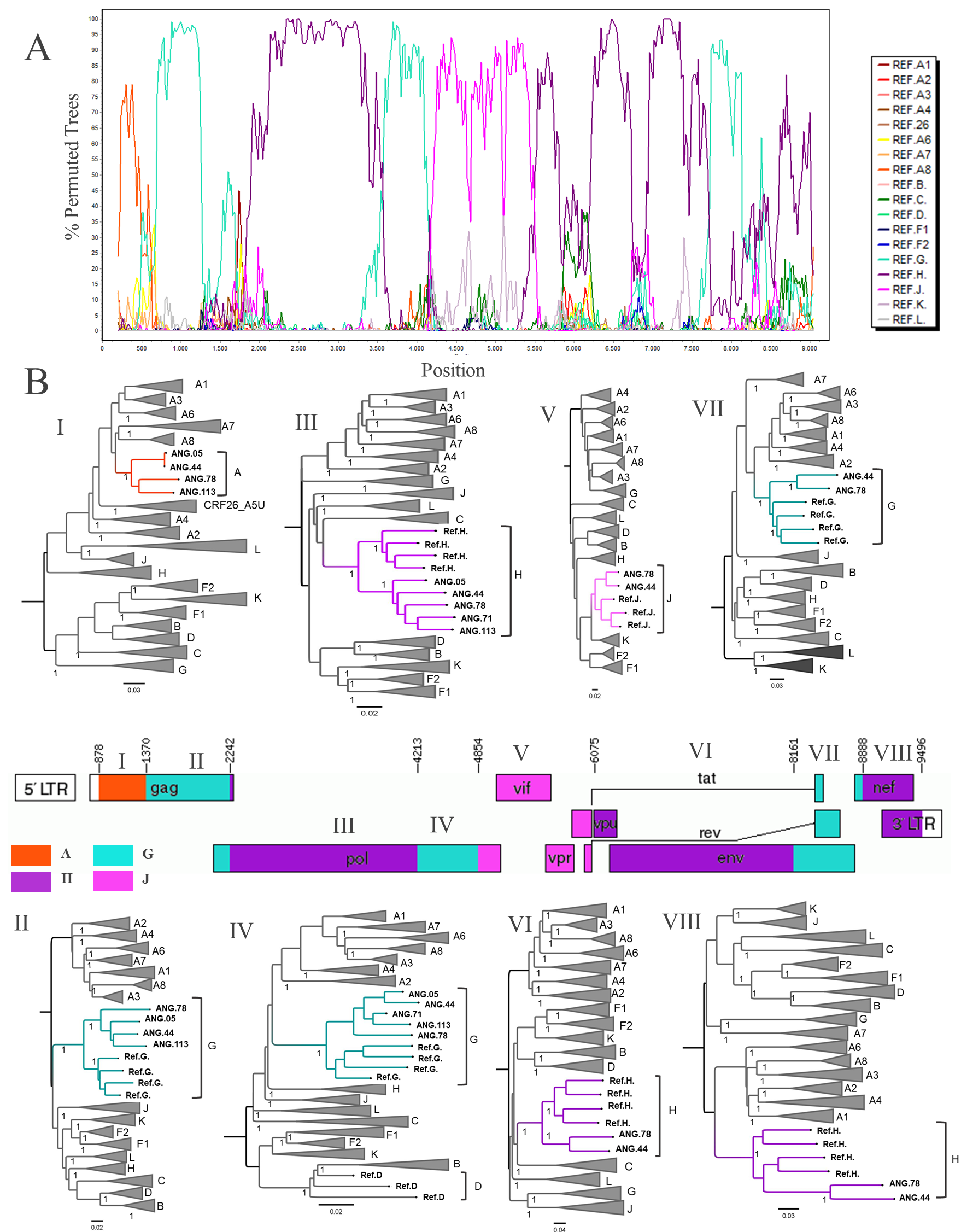

Supplement: Supplementary file 2 [file Image_2.TIF]
