# Supplementary material for: Identification of a new circulating recombinant form of human immunodeficiency virus type 1, CRF124_cpx involving subtypes A, G, H, and CRF27_cpx in Angola
Source: Front Microbiol. 2022 Oct 17;13:992640. doi: 10.3389/fmicb.2022.992640 (PMC9619209; doi:10.3389/fmicb.2022.992640)
Supplement: Supplementary file 3 [file Data_Sheet_1.docx]

**Table S1.** Primers used in this study for nested-PCR and DNA sequencing.

| **Fragment** | **Name of Primers** | **Reactionª** | **Direction** | **Primer Sequence (5’ --> 3’)** |
| --- | --- | --- | --- | --- |
| **A ( 408-2594)** | SCAOSD | PCR 1R | Forward | GGGACTTTCCGCTGGGGACTTTC |
|  | LR51 | PCR 1R | Reverse | GTATTCCTAATTGAACYTCC |
|  | SCANSD | PCR 2R; SEQ | Forward | CGAGCCCTCAGATGCTGCATATAAGC |
|  | DP11 | PCR 2R; SEQ | Reverse | CCTGGCTTYAATTTYACTGGTA |
|  | H1G777AS | SEQ | Reverse | CCATGCRTTYAARGTYCT |
|  | MZ14 | SEQ | Reverse | GAACCKRTCTACATAGTCTC |
|  | H1P202 | SEQ | Reverse | CTAATACTGTATCATCTGCTCCTG |
|  | GAG3 | SEQ | Forward | CTRGGATTAAATAAAATAGT |
| **B (2216-4674)** | LR49 | PCR 1R; PCR 2R; SEQ | Forward | CAATGGCCATTGACAGAAGA |
|  | SCCOAS | PCR 1R | Reverse | TATTCTTTCCCCTGCACTGTA |
|  | MMINT4 | PCR 2R; SEQ | Reverse | CTTGACTTTGGGGATTGTAGGG |
|  | DP16 | SEQ | Forward | CCTCARRTCACTCTTTGGCARC |
|  | MMRT12 | SEQ | Reverse | ATCAGGATGGAGTTCATAMCCCATCCAAAG |
|  | LR51 | SEQ | Reverse | GTATTCCTAATTGAACYTCC |
|  | SEQ-RT | SEQ | Forward | ATGGAAAGGATCACCAGCAA |
|  | MMRT3 | SEQ | Reverse | TGTGCTGGTACCCATG |
|  | MMRT6 | SEQ | Reverse | TTTTACATCATTAGTGTGGG |
|  | MMRT7 | SEQ | Forward | CATGGGTACCAGCACA |
| **C (4173-5213)** | MMRT7 | PCR 1R | Forward | CATGGGTACCAGCACA |
|  | MMINT7 | PCR 1R | Reverse | TCTCCTGTWTGCARMCCCCAATATGTTGT |
|  | MMRT2 | PCR 2R; SEQ | Reverse | ATCAGGATGGAGTTCATAMCCCATCCAAAG |
|  | MMINT6 | PCR 2R; SEQ | Reverse | GGGATGTGTACTTCTGAACTT |
|  | MMINT3 | SEQ | Forward | CCCTACAATCCCCAAAGTCAAG |
|  | MMINT4 | SEQ | Reverse | CTTGACTTTGGGGATTGTAGGG |
| **D (4890-5977)** | SCCOS | PCR 1R | Forward | TACAGTGCAGGGGAAAGAATARTAGACATAATA |
|  | ED3AS | PCR 1R; PCR 2R; SEQ | Reverse | CCTGCCATAGGARATGCCTAA |
|  | SCCNS | PCR 2R; SEQ | Forward | CAAAATTTCCGGGTTTATTACAGGGACA |
|  | VIF1 | SEQ | Forward | ATGGAAAACAGATGGCAG |
|  | VIF2 | SEQ | Forward | TGTTTTKCAGAMTCTGC |
|  | VIF1 | PCR 1R | Forward | ATGGAAAACAGATGGCAG |
| **E (5380-7336)** | JH48 | PCR 1R | Reverse | RATGGGAGGRGYATACA |
|  | VIF2 | PCR 2R | Forward | TGTTTTKCAGAMTCTGC |
|  | ENV04AS | PCR 2R | Reverse | ATTTCTRGGTCCCCTCCTGA |
|  | ED3 | SEQ | Forward | GTTAGGCATYTCCTATGGCAG |
|  | ED5 | SEQ | Forward | ATGGGATCAAAGCCTAAARCCATGTG |
|  | ENV03AS | SEQ | Reverse | TTTAGGCTTTGATCCCAT |
|  | VPR1S | SEQ | Forward | GAAGCTGTYAGACAYTTTCC |
|  | VPR1AS | SEQ | Reverse | GGAAARTGTCTRACGCTTC |
|  | VPU1S | SEQ | Forward | AGAGCAGAAGACAGTGGCAA |
|  | VPU1AS | SEQ | Reverse | TTGCCACTGTCTTCTGCTCT |
| **F (6817-8296)** | ED5 | PCR 1R | Forward | ATGGGATCAAAGCCTAAARCCATGTG |
|  | ENV02AS | PCR 1R | Reverse | TATCCCTKCCTAACTCTAT |
|  | ED31 | PCR 2R; SEQ | Forward | CCTCARYCATWACACARGCYTGTCCAAAG |
|  | MM4 | PCR 2R; SEQ | Reverse | CCTCCTACTATCATTATGAA |
|  | JH48 | SEQ | Reverse | RATGGGAGGRGYATACA |
|  | SCDOS | SEQ | Forward | TTGAACCAYTAGGARTAGCACCCAC |
|  | ED12 | SEQ | Reverse | AGTGCTTCCTGCTGCTCCCAAG |
| **G (7696-9636)** | MM5 | PCR 1R | Forward | TGARRGACAATTGGAGAAGTG |
|  | SCDOAD | PCR 1R | Reverse | AGTCACACAACAGACGGGCACACAC |
|  | SCDOS | PCR 2R; SEQ | Forward | TTGAACCAYTAGGARTAGCACCCAC |
|  | SCDNAD | PCR 2R; SEQ | Reverse | AGGCAAGCTTTATTGAGGCT |
|  |    \| ENV02AS \| \| --- \| | SEQ | Reverse | TATCCCTKCCTAACTCTAT |
|  | TAT1 | SEQ | Forward | ATAGAGTTAGGMAGGGATA |
|  | NEF3 | SEQ | Forward | GRACAGATAGRRTYATAGAA |

a PCR 1R – first round; PCR 2R – second round; SEQ – sequencing. Reference of the primers: (Delwart et al., 1993; Zazzi et al., 1993; Salvi et al., 1998; Heyndrickx et al., 2000; Guimarães et al., 2002; Mackay et al., 2004; Sierra et al., 2005; Niyasom et al., 2009; Passaes et al., 2009; Qu et al., 2010; Delatorre et al., 2017)

**Supplementary figure 1.** Maximum likelihood (ML) genome tree implementing nucleotide substitution model General Time Reversible (GTR), indicating the phylogenetic relationships between HIV-1 subtypes, sub-subtypes A and F and CRF (until CRF118). Approximate likelihood ratio test (ALRT) values are represented only if > 0.90. The Angolan sequences were highlighted in purple.

**Supplementary figure 2.** Bootscan analysis of the sequence ANG.44 was performed in simplot software using a 400 nt sliding window and 20 nt increments. B) Genomic structure was colored according to the HIV-1 subtyping. The mosaic map was generated using the Recombinant HIV-1 Drawing Tool (https://www.hiv.lanl.gov/content/sequence/DRAW_CRF/recom_mapper.html).The maximum likelihood tree (ML) was performed to confirmation of the HIV-1 subtype of each fragment. ML tree implementing nucleotide substitution model General Time Reversible (GTR), indicating the phylogenetic relationships between pure HIV-1 subtypes and analyzed sequences. ALRT values ​​were represented only if > 0.90.

**Table S2.** Prevalence of complete genomic sequences of subtypes A, G, H and J obtained from the Los Alamos database.

| **Complete HIV-1 Genomes** | | |
| --- | --- | --- |
| **Subtype** | **Total of sequences** | **Location** |
| **A** | 303 | Rwanda |
|  | 172 | Russian Federation |
|  | 129 | Kenya |
|  | 104 | Tanzania |
|  | 86 | Uganda |
|  | 27 | Cyprus |
|  | 25 | Pakistan |
|  | 19 | Ukraine |
|  | 11 | Democratic Republic of Congo |
|  | 10 | Sweden |
|  | 9 | India |
|  | 7 | South Africa |
|  | 7 | Uzbekistan |
|  | 6 | Cameroon |
|  | 6 | Kazakhstan |
|  | 6 | Spain |
|  | 6 | United Kingdom |
|  | 3 | Bulgaria |
|  | 3 | Nigeria |
|  | 3 | Senegal |
|  | 2 | Australia |
|  | 2 | Belarus |
|  | 2 | Cape Verde |
|  | 1 | Belgium |
|  | 1 | Georgia |
|  | 1 | Italy |
|  | 1 | Slovenia |
|  | 1 | Switzerland |
|  | **953** | **TOTAL** |
| **G** | 28 | Nigeria |
|  | 18 | Cameroon |
|  | 13 | Spain |
|  | 6 | China |
|  | 6 | Kenya |
|  | 4 | Democratic Republic of Congo |
|  | 4 | Portugal |
|  | 4 | Russian Federation |
|  | 3 | Cuba |
|  | 3 | Ghana |
|  | 3 | United Kingdom |
|  | 2 | Belgium |
|  | 1 | Bulgaria |
|  | 1 | Guinea- Bissau |
|  | 1 | South Africa |
|  | 1 | Sweden |
|  | 1 | - |
|  | **99** | **Total** |
| **H** | 4 | Democratic Republic of Congo |
|  | 3 | Belgium |
|  | 2 | Central African Republic |
|  | 1 | United Kingdom |
|  | **10** | **Total** |
| **J** | 3 | Sweden |
|  | 2 | Democratic Republic of Congo |
|  | 1 | Angola |
|  | 1 | Cameroon |
|  | **7** | **Total** |
